# Using a theory driven approach to develop and evaluate a complex mental health intervention: the friendship bench project in Zimbabwe

**DOI:** 10.1186/s13033-016-0050-1

**Published:** 2016-02-29

**Authors:** Dixon Chibanda, Ruth Verhey, Epiphany Munetsi, Frances M. Cowan, Crick Lund

**Affiliations:** Department of Community medicine, Zimbabwe Aids Prevention Project-University of Zimbabwe, Harare, Zimbabwe; Research Department of Infection and Population Health, University College London, London, UK; Centre for Sexual Health and HIV AIDS Research Zimbabwe, Harare, Zimbabwe; Alan J Flisher Center for Public Mental Health, Department of Psychiatry and Mental Health, University of Cape Town, Cape Town, South Africa

**Keywords:** Theory of change, Mental health, Complex intervention, Stakeholder involvement

## Abstract

**Background:**

There is a paucity of data on how to deliver complex interventions that seek to reduce the treatment gap for mental disorders, particularly in sub-Saharan Africa. The need for well-documented protocols which clearly describe the development and the scale-up of programs and interventions is necessary if such interventions are to be replicated elsewhere. This article describes the use of a theory of change (ToC) model to develop a brief psychological intervention for common mental disorders and its’ evaluation through a cluster randomized controlled trial in Zimbabwe.

**Methods:**

A total of eight ToC workshops were held with a range of stakeholders over a 6-month period with a focus on four key components of the program: formative work, piloting, evaluation and scale-up. A ToC map was developed as part of the process with defined causal pathways leading to the desired impact. Interventions, indicators, assumptions and rationale for each point along the causal pathway were considered.

**Results:**

Political buy-in from stakeholders together with key resources, which included human, facility/infrastructure, communication and supervision were identified as critical needs using the ToC approach. Ten (10) key interventions with specific indicators, assumptions and rationale formed part of the final ToC map, which graphically illustrated the causal pathway leading to the development of a psychological intervention and the successful implementation of a cluster randomized controlled trial.

**Conclusion:**

ToC workshops can enhance stakeholder engagement through an iterative process leading to a shared vision that can improve outcomes of complex mental health interventions particularly where scaling up of the intervention is desired.

## Background

Mental, neurological and substance use (MNS) disorders contribute significantly to the global burden of disease, particularly in low and middle income countries (LMIC) [1], where the largest treatment gap for MNS disorders exists [2]. In recent years scaling up of MNS services has been recommended [3] through the development of packages of care that emphasize task shifting as a way of addressing this treatment gap [3–5]. There is growing evidence suggesting that appropriately trained and supported lay health workers can deliver interventions for MNS disorders in low resource settings [6] with a number of clinical trials showing efficacy of this approach [7–10]. However, there is a paucity of data and capacity on how to deliver such complex interventions in routine primary care settings in a manner that reduces the treatment gap for MNS disorders, particularly in sub-Saharan Africa [11, 12].

The need for well documented protocols which clearly describe the development and the scale-up of programs and interventions is necessary if such interventions are to be replicated elsewhere [13]. In recent years the theory of change (ToC) approach has become widely used as a tool for developing and evaluating complex interventions [14], because of it’s theory driven approach to evaluation [15], ability to facilitate stakeholder participation, explicit identification of causal pathways, and potential for linking indicators to the design of complex interventions [16]. Existing recommended evaluation guidelines for complex interventions such as the Medical Research Council (MRC) [17] framework do not describe the mechanism of change through which a given intervention or program leads to real-world impact [18]. The ToC defines how and why an initiative works through the use of evidence based measures and indicators to explain an initiative’s causal pathway to impact [14].

The process of developing a ToC starts early during an initiative with key stakeholders invited to develop a common vision that describes the causal pathway leading to the program/initiative/intervention goal. During this process, specific outcomes, indicators, assumptions, barriers and interventions are identified to ensure that the desired outcomes are realized [14]. ToC therefore offers a roadmap for the necessary change leading to a desired outcome [19]. This map offers information on assumptions such as the final destination, the context for the map and the process to engage in during the journey. Furthermore, the ToC map outlines the belief system that underlies the steps in the causal pathway, and describes the input and outcome of the different level interaction. A series of meetings and workshops with relevant stakeholders is the main vehicle for developing a theory of change map. Such an approach has been used in the program for improving mental health care (PRIME) [5], a multi-country complex intervention aimed at generating evidence on how to integrate mental health into primary care through the development, implementation and evaluation of district level mental health care plans for priority disorders [16].

Little is known about the use of the Theory of Change approach in the development and evaluation of mental health interventions in the context of HIV in sub-Saharan Africa. There is evidence suggesting that the ToC approach can be a useful tool for developing and evaluating such interventions including establishing evaluation frameworks, and obtaining the necessary buy-in of key stakeholders [14].

We recently utilized a ToC approach to develop, and evaluate through a cluster randomized controlled trial the Friendship Bench project [20], an intervention utilized predominantly by people living with HIV (PLWH). The Friendship Bench has been running in three large primary care clinics in Harare Zimbabwe for over 8 years. It is a task shifting program that uses lay health workers to deliver a structured cognitive behavior therapy (CBT) based intervention that emphasizes problem solving therapy (PST) [21]. A decision to scale-up the intervention to 60 primary care facilities employing over 300 LHWs was recently made by the city health authorities. This paper describes the manner in which the ToC model was used to design and evaluate a successful cluster randomized controlled trial (RCT) and a scale-up plan [20].

## Methods

Eight (n = 8) ToC workshops were held with relevant stakeholders over a 6-month period. Stakeholders included policy makers from the Ministry of Health and Child Care and City of Harare Health Department staff. Table 1 shows the full list of workshop participants by type of workshop and category of participant. Key groups of the stakeholders attended all workshops (City of Harare Health staff, and the research team from the Friendship Bench project).

**Table 1:** Participants by workshop

	Type of workshop				
(n = Number of workshops)	Official launch (n = 1)	Intervention development) (n = 2)	Pilot RCT (n = 2)	Cluster RCT (n = 2)	Scale-up (n = 1)
Total number of participants:	54	28	29	24	14
Policy makers					
Health ministry	8	1	1	0	2
City health	6	2	1	2	2
University lecturer	4	1	1	1	1
Community level workers	6	2	2	2	2
Nurse in charge	8	2	4	2	0
DHPO^a^	2	4	2	2	2
LHW^b^	8	8	2	4	1
Research team	6	3	4	4	
Psychiatrist	3	1	1	1	1
Senior Psychologist	1	1	1	1	1
Psychologist	1		2	2	0
Project coordinator	1	1	2	1	1
Study participants		2	2	0	1
Research assistants	0		4	2	0

^a^District health promoting officer, ^b^ Lay health worker

Invitations to relevant stakeholders were sent out together with the objective of the first meeting. The initial workshop aimed to draw on various sources of information as the first step towards planning for the RCT of the Friendship Bench. This first workshop, which was part of a formal launch of the initiative focused on defining the main components of the process.

### Areas addressed during ToC workshops

Five specific areas related to the Friendship Bench and it’s evaluation through a cluster RCT were addressed during workshops. These included: (a) the perceived impact of the intervention on the care of patients utilizing primary health care facilities particularly PLWH, (b) establishing intermediate and early outcomes which would be arranged on a causal pathway, (c) interventions needed to initiate each of the short, medium, and long term outcomes, (d) the conditions required for each step to be achieved, and (e) the resources required to implement the intervention. Assumptions made during the ToC meetings were based on information gathered over the last 8 years of work carried out on the Friendship Bench in the area of Mbare [22]. We included assumptions that certain conditions would be met in the development of the ToC map as described in previous studies [19].

The first author (DC) facilitated the workshops and constructed the ToC diagrams, while a co-facilitator appointed for each workshop took notes or audio-recorded the meetings. Different workshops for different groups were held to avoid power differentials. Meetings were held at the study sites, University of Zimbabwe Department of Psychiatry, study administrative offices, and City Health Department and lasted between 2 and 4 h. After the first workshop, a sub-group of workshop members continued to work on the ToC under the guidance of the first author DC with scheduled larger workshops including all stakeholders running roughly monthly for 6 months. Informal communication through email, telephone calls, and one on one meetings with specified stakeholders contributed to information that was used in framing the larger group meetings and workshops.

Issues perceived to be important in the development of a successful ToC based on the literature [23, 24] were addressed under four broad themes of (1) Formative work, (2) Piloting, (3) Evaluation, and (4) Scale-up.

#### Formative work (official launch/intervention development)

**Fig. 1:**
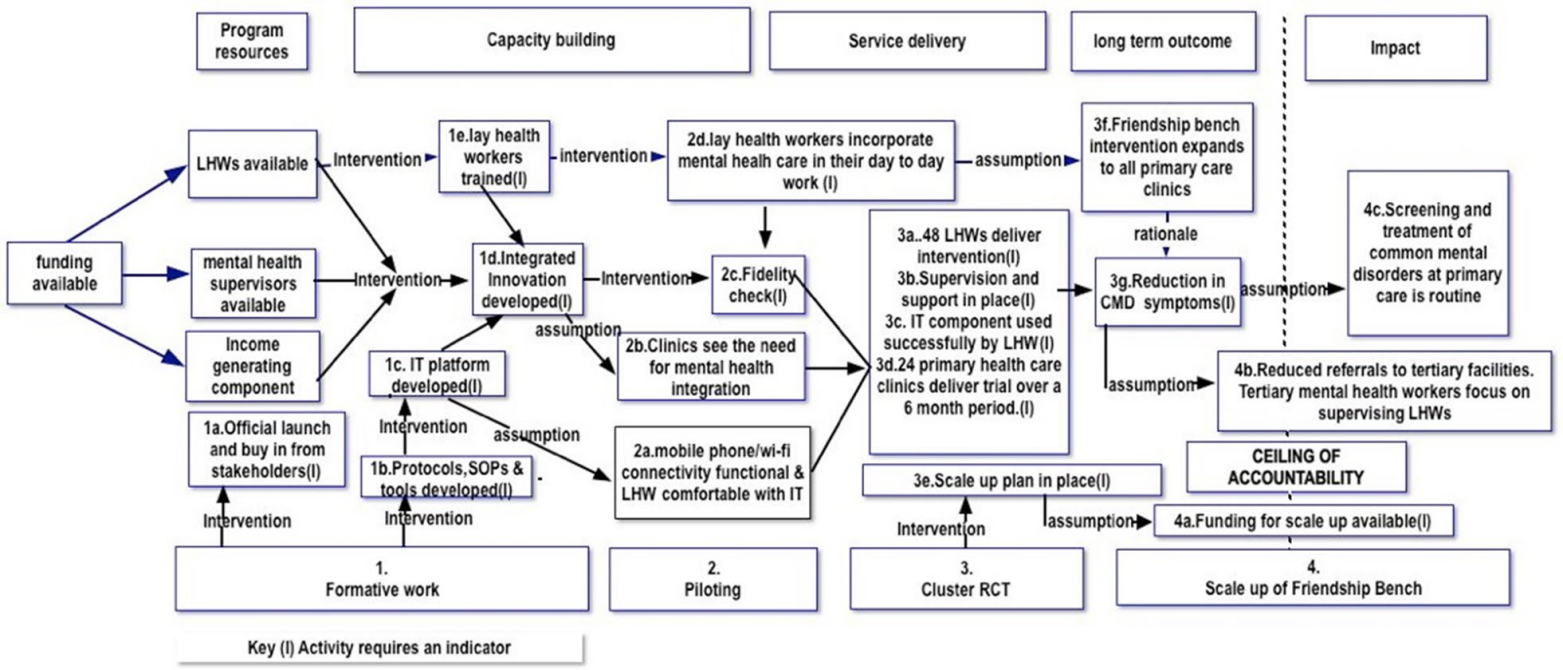
Theory of change map for the friendship bench project. *I* Intervention

The first formative ToC workshop was part of the official launch of the project, which was attended by both the Director of City Health Services, the Minister of Health and several stakeholders described in Table 1. A series of presentations highlighting the need to integrate mental health care into primary care, the evidence, resources available and the processes required were highlighted. These were then discussed with suggestions, comments, from the 54 participants recorded by DC. Through a participatory discussion initiated by the Director of City Health Services and facilitated by DC, broad consensus was reached on the program’s desired impact of having screening and treatment for mental neurological and substance use disorders integrated into all primary health care facilities. Specific issues highlighted during this first ToC workshop included the need to better understand the key components of the intervention and how it would be delivered. Furthermore it was suggested that core competencies for LHWs meant to deliver the PST on the Friendship Bench be established. One follow-up meeting with a smaller group (n = 28) recommended that more information on what the LHWs were capable of doing in terms of workload and competency be collected through the existing site of Mbare [22] and the HIV clinics. The ToC activities related to the formative work are illustrated as 1a–e in the ToC diagram (Fig. 1).

The third workshop focusing on the formative stage included senior clinic managers of the City Health Department, their supervisors, and the Director of City Health services (n = 28). This workshop focused on the role of the clinic nurses and how 24 clinics out of the existing 60 eligible clinics would be selected for the trial. Barriers and challenges to the selection of the 24 clinics were discussed and interventions aimed at clarifying the issue related to these barriers discussed. While the original plan for the program was to utilize clinic-nursing staff to provide supervision to the LHWs at clinic level during the cluster RCT, the ToC workshop noted that nurses were already over-stretched and could not provide supervision and support counseling to LHWs. Therefore there was need to present an acceptable role for the nurses in the 24 cluster RCT clinic sites which was not related to the provision of counseling supervision.

#### Piloting

The pilot ToC workshops illustrated in Fig. 1 as 2a–2d looked at specific assumptions made in relation to running the pilot RCT in four clinic sites. These assumptions were used to develop research questions that were included in the final pilot. Members of staff working at the pilot sites were consulted on issues related to recruitment, reimbursement and referral of critical cases. The integration of mental health into general primary health care programs was considered and obstacles such as lack of supervision, possibility for appropriate referral, availability of medication for MNS conditions and support for LHWs were considered. Workshop participants initially assumed that clinics would embrace the idea of integrating mental health into existing primary care services as was the case in the clinic site of Mbare [22]. However, because of the diversity of clinics the ToC process highlighted the need to explore this assumption further, through individual assessment of the 24 clinics proposed for inclusion in the cluster RCT. Further assumptions included the acceptability of the Friendship Bench by PLWH who often had to deal with stigma and victimization at community level.

Assumptions about the acceptability of asking LHWs to use 8-inch computer “tablets” for communication purposes were discussed and it was concluded that an intervention to establish the barriers and enablers of using technological components for the RCT be carried out. Attending LHWs, however, expressed concern that the $230 8-inch “tablet” computers would expose them to possible mugging, theft, and jealousy from LHWs from clinics not included in the cluster RCT. Instead, they suggested exploring the use of the simple and readily available $30 mobile phones.

#### Evaluation (cluster RCT)

Two workshops focused on the cluster RCT illustrated as 3a–g in Fig. 1, with the first workshop attended by all key stakeholders (n = 24) scrutinizing the results of the two pilot feasibility trials described above. During the workshops a framework to identify interventions that would be feasible to improve the final cluster RCT were considered. The resources, the contextual barriers and facilitating factors for the implementation of the cluster RCT were carefully considered during these workshops. For instance exploring the use of WhatsApp as an alternative form of providing support to the supervisors was specified in the ToC workshop including the consideration of having all study team members and LHWs use one specific mobile phone service provider.

The assumption that the Friendship Bench would expand beyond the 24 clinics included in the trial to the 60 city health clinics throughout the city was highlighted, with the main rationale being that a reduction of CMD symptoms in PLWH utilizing the Friendship Bench would lead to an improvement of other key HIV related outcomes such as adherence. During this workshop, a sub-group consisting of DC, the Director City Health Services and RV was set up to focus on exploring further, critical conditions for scale up including funding opportunities.

#### Scale up

Scale up was defined as the expansion of the Friendship Bench from the original three clinics in Mbare [22], initially to the 12 intervention clinics included in the cluster RCT followed by an expansion to the remaining 48 clinics including the control clinics in the cluster RCT(n = 12) [20]. The scale up is indicated in Fig. 1 as 4a–4c.

Political buy-in from key stakeholders particularly the City Health Department and the Ministry of Health was specified as a critical component for successful scale up. Input, process, output and outcome indicators were consolidated into a visual ToC for the entire program which highlighted the causal pathway to scale up with assumptions and interventions considered along the path. For the purposes of this ToC the “ceiling of accountability” which is the point at which the study team relinquishes control over the possible outcomes of the intervention, and therefore does not employ further indicators to measure those outcomes [14] was set for the ToC map.

### Workshop participants

Through out the process, some unexpected interactions and relationships developed between workshop participants, for instance on several occasions informal meetings over lunch, or coffee between small groups managed to address specific issues resulting in subsequent workshops being more productive and cohesive. The director for district health promoters (DHPOs) commented during one of the meetings that *“All these meetings, particularly the small group meetings have really opened my mind on the need to integrate mental health in all our clinics”.*

### Ethical considerations

Ethical approval was obtained for all interventions carried out to address issues raised during ToC workshops, through the Human Research Ethics Committee of the UCT Health Sciences Faculty (REC Ref: 090/2014 and the Medical Research Council of Zimbabwe (Ref: MRCZ/A/1732). Written informed consent was sought from all participants. The ToC development complied with the requirements of the Declaration of Helsinki [25].

### Data management and entry

All data collected during ToC meetings were managed in accordance with the Medical Research Council of Zimbabwe (MRCZ). Data were collected from a number of sources, including process documentation about the workshops and meetings, existing documentation from the Friendship Bench, email correspondence, telephone communication, formative qualitative work and piloting. The project coordinator was responsible together with the study data clerk for compiling data into folders based on the subject matter. Priority areas from each folder including recommendations were noted and further explored either by contacting facilitators of the respective meeting/workshop or carrying out systematic reviews [26]. Where formal interviews were used, semi-structured interviews were the desired approach [27]. Data was gathered into themes based on content and members of the study team met regularly over the 6 month period to discuss the emerging themes which were then summarized and sent back to the larger group and refined based on the feedback. This process was carried out until consensus was reached on issues such as outcomes, indicators, and interventions required to move from one point to the next.

## Results

A final ToC map (Fig. 1) was developed after a total of eight workshops and ten small group meetings. The map was accompanied by a narrative description of the different components including assumptions, interventions, indicators and rationale for the hypothesized causal pathway to impact.

Getting political buy-in and building capacity particularly among LHWs were two themes highlighted in the ToC map, including the development of an acceptable, user friendly and feasible psychological intervention. A number of interventions were recommended leading to the modification of the existing PST with an emphasis on PLWH [26, 27]. Transparency was emphasized through out the process. For instance all clinic directors, district health directors and representatives of LHWs were invited to the official randomization exercise, which used computer generated random numbers to identify 24 clinics for the cluster RCT and allocate them by either intervention or control arm. During this exercise one of the senior clinic nurses had this to say about the process, *“We are all seeing the selection process clearly so there will be nobody suggesting that clinics were selected on the basis of nurse seniority…..”* The specific components of the ToC including outcomes required to reach impact are further illustrated in the ToC map (Fig. 1).

### Causal pathway

The starting point for the Friendship bench ToC map is the establishment of political buy-in (1a) together with identification of key resources, which included human, facility/infrastructure, communication and supervision resources. The Minister of Health’s participation as guest speaker at the launch was seen as a key step towards obtaining political buy-in. The minister of health stated during the ToC launch that, *“mental health care packages should be integrated in existing primary health care services.”*

The use of existing LHWs (1e), city health clinics and supervisors employed by city health services was critical to ensure sustainability of the program post funding. Furthermore, using LHWs was necessitated by the unavailability of nurses due to their busy schedule. One of the nurses when asked how they cope with patients with mental health issues replied, *“We see a number of mental health cases but we don’t’ have the capacity and the adequate time to provide structured counseling for these patients because we have to take care of everybody else at the clinic.”* An additional key strategy for sustainability was the integration of the Friendship Bench into the University of Zimbabwe’s Department of Community Medicine and Psychiatry. The ToC pathway further indicated the need for protocols and standard operating procedures (SOP) defining the pathway (1b) from community to tertiary facility and stipulated that both community nurses and tertiary level nurses should be in constant communication about such referrals. The establishment of a community liaison/outreach team would contribute towards strengthening the integration of mental health in the work carried out by LHWs (2b, 2d). While availability of functional Internet and mobile phone services (2a) would strengthen supervision and fidelity checks (2c) leading to a successful intervention (3a–d). Scaling up of the Friendship Bench after the RCT (3e) would depend on availability of funding (4a), which could lead to the expansion of the initiative to all primary care clinics (3f) and a reduction of CMD among patients utilizing local clinics (3g).

### Intervention

The nature and design of interventions required to ensure smooth flow from one outcome to another were different for the four components described above (Fig. 1). While ample evidence from previous work on the Friendship Bench [22] supported the feasibility and acceptability of the initiative, the acceptability outside the pilot sites was a leading assumption on the ToC map (2b). Furthermore, the views, buy-in and input of patients particularly PLWH were identified as a requirement to move to the next stage in the causal pathway. A person living with HIV contributed the following about acceptability of the Friendship bench, *“The Friendship bench is a good place to go to because the LHWs who run the friendship bench reside in our community and they have visited our homes for other health related issues so they they kind of already know our status (HIV*+*) so I feel comfortable with them.”*

### Assumptions

Our ToC included several assumptions that were deemed necessary to be in place in order for the outcomes to be achieved; these included complete political buy-in throughout the entire initiative and adequate funding particularly for the scale-up to the 60 clinics. The ToC further assumed that all the 60 clinics would appreciate the need for mental health integration leading to an expansion of the initiative (2b), and that the use of a technological platform to provide supervision and support would be acceptable (2c). A major assumption made by the study team, that LHWs would be receptive of using computer tablets after appropriate training, was dispelled when several of the LHWs showed concern about having the 8-inch computer tablets. One LHW had this to say about the 8-inch computer tablets, *“You see, for old people like me to move around with shiny fancy gadgets like these, people will place a spell on me for having this, and also I will be a target with the thieves.”*

For all levels of human resources involved in the initiative, the ToC stipulated that these had to be from existing systems such as the City Health Department, Ministry of Health and University.

### Indicators

The ToC outlined several indicators, which included input, process, output, and outcome indicators. The critical input indicators were political buy-in, which included the availability of a formal support document from the Director of City Health and the Minister of Health as evidence for buy-in (1a), and programme resources including funding. Process indicators included capacity building, fidelity measurement through the analysis of audio-recorded sessions (2c), adherence to standard operation procedures (SOPs), and referral pathways of study participants. Output indicators included use of mobile devices for supervision and support, screening, recruitment, treatment and follow-up. Outcome indicators included the study primary and secondary outcome measures which were reduction in symptoms of common mental disorders, depression, and generalized anxiety disorder at 6 months as described in the cluster RCT protocol.

### Rationale

The key rationale of our ToC causal pathway to impact was that the intervention would lead to a reduction of CMD symptoms among those receiving care through the Friendship Bench RCT (3g).

## Discussion

This study shows how the use of ToC workshops with a focus on enhancing stakeholders’ engagement through an iterative process contributed to the strengthening of key components of a complex intervention. Our ToC workshops systematically addressed four inter-related steps along the causal pathway which consisted of: formative work, piloting, evaluating and implementation of a complex mental health intervention.

Several researchers in the field recommend the use of ToC workshops for the design of complex interventions [14, 23, 28, 29]. Our emphasis on an interactive approach during all the ToC workshops contributed to several positive outcomes including the building of rapport and enhancing stakeholder engagement during the 4 year period of the study. Key stakeholders involved in the ToC workshops were researchers, policy-makers, clinic staff, community health workers (LHWs) and user groups.

Service providers such as nurses, clinic administrators, LHWs and their supervisors provided detailed contextual information which resulted in the final study protocol being revised four times before submission for ethical approval. Some issues raised by service providers during the workshops included the risks associated with providing LHWs with tablet computers (2a), the need to use mobile phone money deposit as a way of reimbursement for study participants, and the need for all clinic staff to be invited to the randomization of clinics so as to avoid tension from clinics that would be excluded from participation in the study as a result of not being randomly allocated.

As has been recommended in previous ToC protocols [14, 16, 23] our initial ToC workshop commenced with a description of the desired impact and building consensus around it (1a). We then used an iterative approach going through the causal pathway by identifying barriers, interventions, formulating rationale and making assumptions about the pathway leading to impact. Sub-groups were formed to avoid tension and hierarchical approach to workshops as has been recommended in similar ToC workshops [16, 29, 30]. There is evidence indicating that hierarchical structures and division of labour found in many organizations often produce a differentiation of decision makers, implementers, and program users [31]. We therefore ran the ToC in groups that were less hierarchical based on these dynamics. This formal consensus development method which involved learning across disciplines has been described as yielding effective results in multidisciplinary mental health teams [32], through the collective selection of strategies that can be applied to promote change [31].

Understanding why and how a complex intervention works contributes significantly to it’s effectiveness, sustainability, and scalability through the provision of evidence based explanations of the mechanisms of change [14]. The ToC workshops for the Friendship Bench managed to provide a clear explanation of the mechanisms of change through the development of the ToC map which gave a clear visual pathway of the study. This enabled all workshop participants regardless of level of research, monitoring and evaluation expertise to have an understanding of the program theory and the implementation theory as described by Weiner [31].

The implementation theory defines the how and why of implementation activities such as planning, training, and resource allocation and how they are linked together to generate the desired results [33]. By providing every workshop participant with a graphic representation of the causal pathway, all were able to understand the need for specific interventions and indicators through the causal pathway. This helped to answer the question “How will we know we are ready to move to the next step?”

While nursing staff at local clinics were reluctant to provide counselling supervision to LHWs, their ability to participate in the workshops and sub-group meetings contributed to their appreciation of both the program theory and implementation theory. This contributed to the overall success of the trial as they facilitated interviewing space, recruitment, and the prescription of anti-depressants to participants who were severely depressed before they referred them to the tertiary mental health services for further management.

Although the use of ToC in mental health programs is in it’s infancy [14, 16], it’s use in settings with complex bureaucratic procedures can be of great value as it brings together stakeholders, increasing the probability that such complex interventions will be more successful. Policy makers often require simple and easy to understand processes which they can run through without needing expert opinion.

## Limitations

There are several limitations to the development of this ToC which include the prominence of the first author (DC) and the Friendship Bench team in most of the activities leading to the desired outcome. This could have contributed to a social desirability bias. Secondly, the analysis of the data from the workshops was carried out by the Friendship Bench team under the guidance of the project coordinator (EM), this too may have contributed to a bias. However, due to the need for stakeholders to stay focused on the ToC process over a 6 month period, the principle investigator (DC) was seen as the most appropriate person to drive the process as he was the only team member familiar with the ToC approach. Furthermore, other stakeholders, although keen to participate in the ToC activities were reluctant to take on the responsibility of leading the process. Another limitation was the minimal involvement of service users in some of the ToC workshops due to the lack of funds to transport them to workshop venues.

Despite these limitations, key lessons learnt from the ToC approach include the need to have a committed person responsible for putting together the different results from the ToC workshops and sharing these with the larger group through an iterative process. The importance of the visual ToC map which can be the most unifying component of the exercise bringing together participants including LHWs with minimal education to contribute towards describing outcomes, interventions, and indicators.

## Conclusion

The use of ToC workshops in the development, piloting, evaluation and implementation of complex interventions in mental health should be encouraged as this increases the likelihood of successful implementation through a combined use of theory and practice and the development of a more transparent causal pathway.
